# Evaluation of genetic relationship between 15 bamboo species of North-East India based on ISSR marker analysis

**DOI:** 10.22099/mbrc.2018.28378.1303

**Published:** 2018-03

**Authors:** Thoungamba Amom, Leimapokpam Tikendra, Hamidur Rahaman, Angamba Potshangbam, Potshangbam Nongdam

**Affiliations:** Department of Biotechnology Manipur University, Canchipur, Imphal-795003, Manipur, India

**Keywords:** Bamboos, genetic diversity, ISSR markers, Bambusa, Dendrocalamus

## Abstract

The classification of bamboos based on floral morphology and reproductive characters is very hard due to erratic flowering behavior and unusually long reproductive cycle. The application of reliable and effective DNA molecular markers is highly essential to address this problem. In the present investigation, inter-simple sequence repeats (ISSR) markers were employed to study phylogenetic relationship of 15 different bamboos of North-East India. The 10 ISSR primers generated 116 polymorphic loci or scorable bands with average of 11.6 bands per primer. The genetic similarity coefficient ranged from 0.41-0.76 showing high genetic polymorphism among different bamboo species. The phylogenetic tree constructed based on genetic similarity matrix revealed genetic proximity of endemic *Bambusa mizorameana* to other five *Dendrocalamus* species by clustering into a single group, while *Dendrocalamus manipureanus* segregated from the cluster indicating its genetic divergent character. Except for *Schizotachyum fuchsianum,* the three *Schizotachyum *species viz., *S. dullooa, S. pergracile* and *S. munroi* exhibited close genetic affinity by grouping into a single minor clade. Principal coordinates analysis (PCoA) showed distribution of different bamboos species in the plot in accordance to unweighted pair-group method with arithmetic average (UPGMA) cluster analysis. Genetic relationship of 15 different bamboos as revealed from the dendrogram and PCoA analysis reasonably conformed to traditional system of classification though slight disagreement existed. This is the first report on the successful use of ISSR markers in the phylogenetic and genetic variation studies of 15 important bamboos of the region including the endemic bamboo species of *B. mizorameana*, *B. manipureana*, *D. sikkimensis* and *D. manipureanus*.

## INTRODUCTION

Bamboos are one of the most important forest trees having more than 1000 different uses [1]. They are used extensively in paper, handicraft and furniture industry, house construction, making water pipes, storage vessels and other household items [2]. Because of their high domestic and commercial utility, proper taxonomy and identification of bamboos are becoming highly essential. The authentic identification of bamboo taxa is also necessary to ensure protection of intellectual property right for breeders and also for commercial propagators and domestic consumers. But identification and genetic relationship study of natural bamboos are daunting tasks due to general lack of morphological differences and erratic flowering pattern [3]. Generally bamboo identification is dependent mostly on vegetative characters like culm and culm sheath morphology due to their abnormally long sexual cycle and absence of any diagnostic tools [4]. However, the vegetative characters are considered less reliable for taxonomic and systematic identification of species as they can be easily influenced by several environmental factors [5]. Also, the taxonomic grouping based on morphological characters is less dependable due to involvement of smaller number of genes for morphological traits which may not reflect truly the scenario of entire genome [6]. 

The traditional method of classification has many shortcomings which can be replaced by new molecular approaches [7]. Many economically important bamboos of Asia and the Pacific regions are not properly identified and taxonomic studies on bamboos are limited as compared to other grass family [8]. Determination of phylogenetic relationship and genetic diversity of available plant germplasm is important for identifying the potential germplasm groups and optimizing hybridization, selection procedures and conservation [9]. Isozyme or DNA markers can be employed for effective genetic identification and differentiation of many plants [10-13]. Isozyme and random amplified polymorphic DNA (RAPD) markers have been used in genetic variation and phylogenetic relationship study among different bamboos [9, 14-16]. Restriction fragment length polymorphism (RFLP) and amplified fragment length polymorphism (AFLP) markers have also been utilized in genetic variation study of *Phyllostachys* bamboos and other four genera within the sub tribe Bambusinae [17 and 18]. However, ISSR primers are more commonly used than RFLP and AFLP markers as they are fast, reliable and require no sequence information and very less quantity of DNA [19 and 20]. ISSR markers can perform effective genetic differentiation at inter and intra specific levels because of their more specific and reproducible amplifications of genomic regions [21]. The distribution of ISSR throughout the genome also allows the amplification of genomic DNA in much higher number of fragments per primer as compared to RAPD markers [22]. Though ISSR markers have been reportedly used in population genetic studies of some bamboos [23-25], their application in molecular phylogenetic and genetic differentiation studies are very limited. 

The North-East India harbors rich bamboo resources as it contributes more than 60% of bamboos in India. 15 important bamboos of the region (*Bambusa tulda*, *B. nutan, B. mizorameana, B. vulgaris*, *B. manipureana, Schizotachyum dullooa*, *S. pergracile*,* S. munroi*,* S. fuchsianum, Dendrocalamus giganteus*, *D. hamiltonii*,* D. sikkimensis*, *D. hookeri*, *D. longispathus *and *D. manipureanus*) have been selected for the present study. Presently the grouping of these bamboos is mostly performed using the traditional classification system. The more sophisticated molecular marker approach has not been effectively utilized to establish genetic and phylogenetic relationship of the local bamboos. The present investigation was carried out with an aim to determine the genetic diversity and relationship between the 15 different bamboos of North-East India using ISSR markers. 

## MATERIALS AND METHODS

**Plant materials and DNA extraction:** Fresh leaf samples obtained from 15 bamboo species namely *Bambusa tulda*, *B. nutan*, *B. mizorameana*, *B. vulgaris*, *B. manipureana, Schizotachyum dullooa*,* S. pergracile*, *S. munroi*,* S. fuchsianum, Dendrocalamus giganteus*,* D. hamiltonii*, *D. sikkimensis*, *D. hookeri*, *D. longispathus *and *D. manipureanus *have been used for extraction of DNA using cetyltrimethylammonium bromide (CTAB) method with some modifications [26]. The leaves were finely ground to fine powder in liquid nitrogen and were mixed with freshly prepared CTAB extraction buffer. The mixture was incubated at 50^o^C for 15-20 minutes in hot water bath before being subjected to centrifugation at 12000rpm for 5 minutes. The supernatant was collected and treated with chloroform: isoamylalcohol (24:1) followed by another centrifugation at 13000 rpm for 1-2 minutes. The pellet obtained after 7.5M ammonium acetate treatment was washed several times with 70% ice cold ethanol and dried before being resuspended in sterile DNase free double distilled water. The DNA solution was incubated at 65^o^C for 20 minutes to destroy any DNase which may be present in it. The extracted DNA solution was finally stored at 4^o^C for subsequent analysis. DNA quality and quantity were determined through spectrophotometry at 260 and 280 nm respectively. The purity and integrity of DNA was tested by performing 1.0% agarose gel electrophoresis and comparing the intensity of the resultant bands with 1kb DNA ladder (Hi Media, India). The DNA samples obtained after extraction process were finally diluted to 50ng/µl and stored at-20^o^C for ISSR marker analysis.

**ISSR amplification:** Out of the 21 ISSR primers initially screened, 10 ISSR primers (UBC-810, UBC-813, UBC-814, UBC-820, UBC-822, UBC-823, UBC-824, UBC-827, UBC-828 and UBC-830) were selected based on production of clear, reproducible and scorable bands. The amplification was carried out in a total volume of 25µl containing 20ng of genomic DNA, 2.5µl of 10X PCR buffer containing 15mM MgCl_2_, 0.2mM dNTPs, 1 unit Taq polymerase (Bangalore Genie, India) and 20ng of ISSR primers (Integrated DNA Technologies, India). The ISSR amplification cycles consisted of an initial DNA denaturation at 94^o^C for 4 mins followed by 45 cycles of denaturation at 94^o^C for 1 min, annealing at 40^o^C for 1min and 2mins extension at 72^o^C with a final extension at 72^o^C for 10 mins. Thermal cycler (Hi Media, India) was used for DNA amplification and amplified fragments generated were separated on 1.5% (w/v) agarose gel using 1 X TBE buffer. The gel with the separated fragments was stained with ethidium bromide (0.5µg/ml) and finally visualized and photographed under Gel Documentation system (Syngene, UK). A 1kb DNA ladder (Hi Media, India) was used to determine the size of unknown DNA fragments on the agarose gel. The amplification reactions of ISSR primers were performed twice to ensure the reproducibility of the banding pattern.

**Data analysis: **Distinct and well resolved bands were scored as present (1) or absent (0) for each ISSR reaction. The data obtained were utilized to compile binary data matrix and the intensity of band was not considered while scoring. The genetic similarity matrix between 15 different bamboo species was calculated according to Nei and Li [27]. These similarity coefficients obtained were used to construct Neighbor-Joining dendrogram through Mega 5.10 software [28] using unweighted pair-group method with arithmetic average (UPGMA) method. The Principal coordinate analysis (PCoA) was employed to determine the spatial representation of genetic distances observed among different bamboo species and also to check the consistency of genetic differentiation as defined by cluster analysis. Principal coordinate analysis (PCoA) was performed using GenALEx version 6.5 [29]. 

## RESULTS AND DISCUSSION

**Figure 1:**
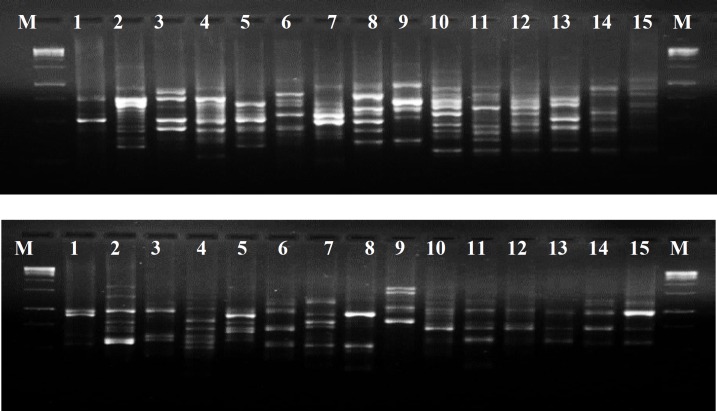
(A) ISSR marker profile of 15 different bamboo species using UBC-810 primer, (B) ISSR banding profile of the 15 bamboo species using UBC-823 primer. M represents 1000bp DNA ladder marker and lanes 1-15 are template DNA each derived from individual species of *Bambusa tulda*, *B*. *nutan, B*. *mizorameana*, *B. vulgaris*, *B. manipureana*, *Schizotachyum dullooa*, *S. pergracile*, *S*. *munroi*, *S. fuchsianum*, *Dendrocalamus giganteus*, *D*.* hamiltonii*, *D*. *sikkimensis*, *D*. *hookeri*, *D. longispathus *and *D. manipureanus*, respectively

The 10 ISSR primers selected from the 21 initially screened ISSR primers produced a total of 116 scorable bands or loci with average of 11.6 bands per primer (Table 1). The size of amplification fragments ranged from 250 to 2000 bp. UBC-810 produced the highest number of bands (20) while the least (5) was generated by UBC-830. The ISSR banding profiles of UBC-810 and UBC-823 with 15 bamboo species are depicted in Figure 1. The ISSR primers yielded 116 polymorphic loci to estimate phylogenetic relationship between the different bamboo species. which was adequate for effective genetic differentiation. Zhou et al [30] also obtained the stable and reliable information of genetic variation between species when the locus number exceeded 70. Nei [31] observed that a minimum of 50 different loci was essential for determining genetic distances between different species. Nayak et al. [9] also recorded a total of 137 polymorphic loci from 10 RAPD primers while analyzing genetic variation studies of 12 different bamboo species.

**Table 1:** Different ISSR primers used with their sequences and number of polymorphic bands produced during amplification with band size

**Primer code**	**Primer sequence**	**No of scorable bands**	**No of** **polymorphic bands**	**Degree of polymorphism (%)**	**Amplification fragment size (bp)**
UBC 810	5’-*GAGAGAGAGAGAGAGAT*-3’	20	20	100	250-1500
UBC 813	5’-CTCTCT CTCTCT CTCTT- 3’	12	12	100	350-1500
UBC 814	5’- CTCTCTCTCTCT CTCTA-3’	12	12	100	250-1500
UBC 820	5’- GTGTGTGTGTGT GTGTC-3’	10	10	100	500-2000
UBC 822	5’ -TCT CTCTCTCTCTCT CA-3’	13	13	100	350-2000
UBC 823	5’ -TCT CTCTCTCTCTCT CC-3’	12	12	100	350-2000
UBC 824	5’- TCT CTCTCTCTCTCT CG-3’	8	8	100	500-1500
UBC 827	5’- ACA CACACACACACA CG-3’	12	12	100	350-1500
UBC 828	5’- GTGTGTGTGTGT GTGTA- 3’	12	12	100	250-1200
UBC 830	5’- TGT GTGTGTGTGTGT GG-3’	5	5	100	500-1000
Total-10	116				

The genetic similarity index between 15 different bamboo species derived from Nei and Li’s coefficient [27] ranged from 0.41 to 0.76 (Table 2). High genetic proximity was shown between *Dendrocalamus hamiltonii* and *D*. *sikkimensis* (0.76), *D. hookeri* and *D*. *longispathus* (0.75), *Bambusa nutans* and *B. manipureana* (0.72) and *Schizotachyum pergracile *and *S. munroi *(0.63), while low genetic affinity was observed between *Schizotachyum pergracile *and* Dendrocalamus longispathus *(0.41), *S. dullooa *and* D. manipureanus *(0.44), *D. hamiltonii *and *S. pergracile *(0.46) and *D. hookeri* and *S. pergracile *(0.46). 

**Figure 2:**
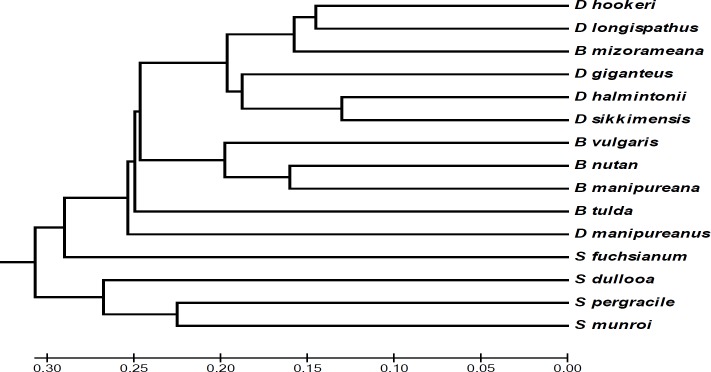
UPGMA dendrogram based on Nei and Li’s coefficient of similarity matrix of 15 different bamboos of North- East India

The dendrogram generated based on similarity matrix obtained from differential banding profile of ISSR primers revealed two major clusters (Fig. 2). The first cluster comprised of members belonging to genus *Schizotachyum *(*S. dullooa*,* S. pergracile *and* S. munroi*)*, *while the remaining 12 bamboos were grouped into other major cluster. Interestingly *S. fuchsianum* was placed separately from the first cluster which indicated its less genetic affinity with other three *Schizotachyum* bamboos. *S. fuchsianum* exhibited lower genetic similarity with *S. dullooa* (0.57), *S. pergracile* (0.55) and *S. munroi* (0.53) as compared to similarity coefficient recorded between *S. dullooa* and *S. pergracile* (0.62), *S. munroi* and *S. pergracile* (0.63) and *S. dullooa* and *S. munroi* (0.61). 

**Table 2:** Genetic similarity matrix between 15 bamboo species based on Nei and Li’s coefficient of similarity.

	1	2	3	4	5	6	7	8	9	10	11	12	13	14	15
1	1.00														
2	0.65	1.00													
3	0.69	0.63	1.00												
4	0.61	0.66	0.66	1.00											
5	0.65	0.72	0.62	0.68	1.00										
6	0.59	0.59	0.56	0.60	0.62	1.00									
7	0.56	0.56	0.56	0.54	0.56	0.62	1.00								
8	0.51	0.53	0.48	0.50	0.58	0.61	0.63	1.00							
9	0.58	0.58	0.50	0.50	0.55	0.57	0.55	0.53	1.00						
10	0.55	0.58	0.62	0.57	0.60	0.56	0.55	0.62	0.60	1.00					
11	0.55	0.56	0.69	0.50	0.60	0.50	0.46	0.56	0.55	0.65	1.00				
12	0.56	0.57	0.61	0.51	0.62	0.56	0.49	0.50	0.54	0.71	0.76	1.00			
13	0.63	0.70	0.74	0.64	0.74	0.59	0.46	0.55	0.55	0.65	0.72	0.69	1.00		
14	0.64	0.66	0.71	0.56	0.62	0.51	0.41	0.54	0.57	0.58	0.73	0.67	0.75	1.00	
15	0.56	0.62	0.58	0.52	0.65	0.44	0.48	0.51	0.60	0.56	0.62	0.61	0.63	0.62	1.00

The 12 bamboos placed under one major cluster were further grouped into different subclusters. Out of the 6 *Dendrocalamus* bamboos included in the study, 5 were grouped in the same subcluster divulging closer genetic identity between them. This finding affirmed their placing under the same genus *Dendrocalamus* as per the traditional system of classification (Fig. 2).

The lone *D. manipureanus* was genetically more divergent from other *Dendrocalamus* species as it was placed separately by segregating from the group. The total segregation of *D. manipureanus* from other members of *Dendrocalamus* indicated the more likely polyphyletic origin of the genus *Dendrocalamus*. Ghosh et al. [32] using AFLP approach also revealed the formation of distinct cluster of the landraces of *D. manipureanus* and their segregation from *D. latiflorus*. The general morphological features of *D. hookeri* and *D. longispathus* are closely similar so it is difficult to differentiate their natural stands [33]. The dendrogram representation obtained in the present study also revealed the clustering of these two *Dendrocalamus* bamboos into a single minor clade supporting their close phenotypic and genetic relationship. Similar explanation can be made for *D. giganteus* and *D. sikkimensis* which exhibited identical morphological features and were placed in same group. *D. giganteus* was also placed in same subcluster with *D. hamiltonii*. Basumatary et al. [34] while studying the phylogenetic relationship of 15 tropical bamboos using 35 morphological descriptors (MD) also grouped *D. giganteus* and *D. hamiltonii* in the same cluster. Many similar phenotypic characters are shared between *D. giganteus* and *D. longispathus* with their erect culms which are about 14-25m tall and 10-12cm in width with prominent nodes and internodes [33]. But the two *Dendrocalamus* bamboos showed considerable genetic divergence with similarity index of 0.58 recorded between them and occurring in different minor clades in the dendrogram. Ramanayake [8] also reported similar observation of genetic diversity between *D. longispathus* and *D. giganteus* when RAPD analysis was performed to estimate their phylogeny. 

The 3 *Bambusa* species viz., *B. vulgaris, B. nutans *and* B. manipureana* were grouped in same subcluster depicting genetic proximity between them. Sun et al. [35] also reported clustering of *Bambusa nutans* and *B. vulgaris* while studying phylogenetic relationship using RAPD markers. *B. tulda* was however segregated from the group indicating lesser genetic affinity with other 3 members of *Bambusa*. This result was in contrast to previous report of close genetic affinity between *B. tulda* and *B. vulgaris* by Loh et al. [18] when they studied genetic relationship of bamboo subtribe Bambusinae using AFLP markers. However, in line with our finding Desai et al. [36] also revealed less genetic closeness between *B. tulda* and *B vulgaris* when they recorded Jaccards’s similarity coefficient of only 0.224 using ISSR markers. Genetic similarity matrix analysis revealed *B.mizorameana* to be more genetically closed to *D. hookeri* and *D. longispathus* than to other *Bambusa* species. This is reflected in phylogenetic tree with *B. mizorameana* clustering in same group with *D. hookeri* and *D. longispathus* rather than with other *Bambusa *species. 

The present phylogenetic study showed reservation in the positioning of *B. mizorameana* under *Bambusa* and suggested the appropriateness of *B.mizorameana* to be included within the genus of *Dendrocalamus*. There is still a requirement of in-depth assessment for the accurate classification of two endemic bamboos of *B. mizorameana and* *D. manipureanus. *This is because the normal traditional classification system of bamboos is largely based on morphological and vegetative characters which need to be further validated with the application of other sophisticated and more informative DNA markers [18].

**Figure 3:**
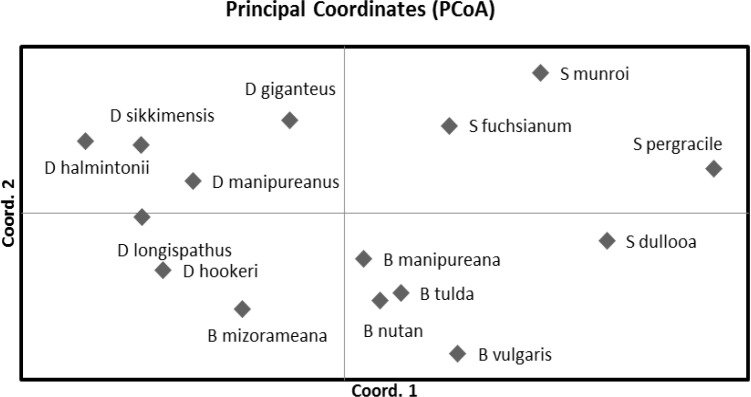
Two dimensional plot of Principal coordinate analysis (PCoA) showing clustering of 15 different species of bamboos

The two dimensional PCoA plot showed the first principal coordinate accounting for 19.19% while the second coordinate produced 14.39% of the total genetic variation. PCoA analysis revealed each bamboo species forming a separate plot and all the species were distributed in agreement with the UPGMA cluster analysis (Fig. 3). The 15 bamboo species were classified into two groups along the axis 1 which separated the 6 *Dendrocalamus* species from the rest represented by *Schizotachyum *and *Bambusa* species. 

The phylogenetic relationship established between 15 bamboo species of North-East India using ISSR markers was quite reasonably conformed to traditional system of classification though some slight discord existed. This minor disagreement with classical approach was also previously observed in genetic relationship study of other bamboo species [8, 9, 15, 37]. This study reports for the first time the use of ISSR markers in the genetic variation and phylogenetic investigation of 15 different bamboos of the region including the four endemic species of *B. mizorameana*, *B. manipureana*, *D. sikkimensis* and *D. manipureanus*.
